# Supplementary material for: Degradation of blue-phosphorescent organic light-emitting devices involves exciton-induced generation of polaron pair within emitting layers
Source: Nat Commun. 2018 Mar 23;9:1211. doi: 10.1038/s41467-018-03602-4 (PMC5865184; doi:10.1038/s41467-018-03602-4)
Supplement: Supplementary file 1 — Supplementary Information(PDF 2094 kb) [file 41467_2018_3602_MOESM1_ESM.pdf]

Supplementary Information for

Degradation of blue-phosphorescent organic light-emitting devices involves exciton-induced generation of polaron pair within emitting layers

Sinheui Kim[1], Hye Jin Bae,[2] Sangho Park,[2] Wook Kim,[3] Joonghyuk Kim,[2] Jong Soo Kim,[2] Yongsik Jung,[2] Soohwan Sul,[2] Soo-Ghang Ihn,[2*] Changho Noh,[2] Sunghan Kim,[2*] and Youngmin You[1*]

[1] Division of Chemical Engineering and Materials Science, Ewha Womans University, Seoul 03760, the Republic of Korea

[2] Samsung Advanced Institute of Technology, Samsung Electronics Co., Ltd., Suwon-si, Gyeonggi-do 16678, the Republic of Korea

[3] Department of Electronic Materials, Samsung SDI Co., Ltd., Suwon-si, Gyeonggi-do 16678, the Republic of Korea

CONTENTS

Supplementary Figure 1. Determination of the reduction potential of H S3
Supplementary Figure 2. Stern–Volmer analyses S4
Supplementary Figure 3. Comparison of the rates of photolysis S5
Supplementary Figure 4. Mass analyses of the degradation products in solutions S7
Supplementary Figure 5. Mass analyses of the degradation products in films S8
Supplementary Figure 6. Comparison of the photoluminescence decay rates S9
Supplementary Figure 7. ^{1}H NMR (600 MHz, CD_2Cl_2) spectrum of Ir1 S10
Supplementary Figure 8. $^{13}C\{^{1}H\}$ NMR (150 MHz, CD_2Cl_2) spectrum of Ir1 S11
Supplementary Figure 9. High performance liquid chromatogram for Ir1 S12
Supplementary Figure 10. ^{1}H NMR (600 MHz, CD_2Cl_2) spectrum of Ir2 S13
Supplementary Figure 11. $^{13}C\{^{1}H\}$ NMR (150 MHz, CD_2Cl_2) spectrum of Ir2 S14
Supplementary Figure 12. High performance liquid chromatogram for Ir2 S15
Supplementary Figure 13. ^{1}H NMR (600 MHz, CD_2Cl_2) spectrum of Ir3 S16

Supplementary Figure 14. $^{13}C\{^1H\}$ **NMR (150 MHz, CD_2Cl_2) spectrum of Ir3** S17

Supplementary Figure 15. High performance liquid chromatogram for Ir3 S18

Supplementary Figure 16. ^1H **NMR (600 MHz, CD_2Cl_2) spectrum of Ir4** S19

Supplementary Figure 17. $^{13}C\{^1H\}$ **NMR (150 MHz, CD_2Cl_2) spectrum of Ir4** S20

Supplementary Figure 18. High performance liquid chromatogram for Ir4 S21

Supplementary Table 1. The k_q and K_a values obtained from the Stern−Volmer Analyses shown in Supplementary Fig. 2 S22

Supplementary Table 2. Electroluminescence data for the devices involving emitting layers of H:Ir S23

Supplementary Table 3. Electroluminescence data for the devices involving emitting layers of mCBP:Ir S24

Supplementary Table 4. Electrochemical potentials of H and mCBP S25

Supplementary Reference S36

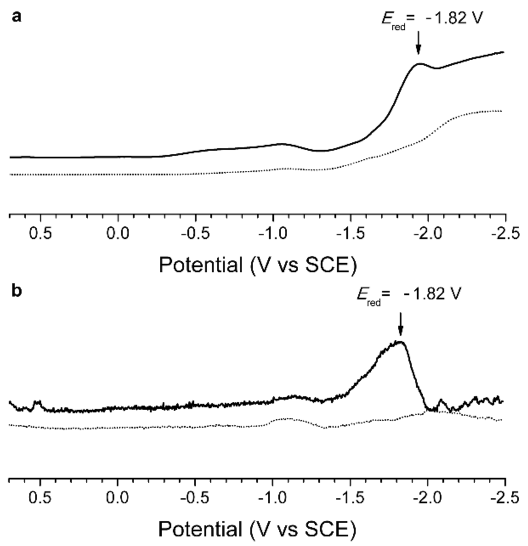

Supplementary Figure 1. Determination of the reduction potential of H. Differential pulse voltammogram (**a**, scan rate = 4.0 mV s^{-1}) and second harmonic alternating current voltammogram (**b,** scan rate = 25 mV s^{-1} and frequency = 100 Hz) of 2.0 mM H dissolved in an Ar-saturated THF solution (2.0 mL) containing 0.10 M $TBAPF_6$ electrolyte. The H solution was delivered to a standard three-electrode cell assembly equipped with a Pt wire counter electrode, a Pt disc working electrode, and an $Ag/AgNO_3$ pseudo reference electrode. The dotted lines in **a** and **b** are the voltammograms of blank solutions (i.e., no H).

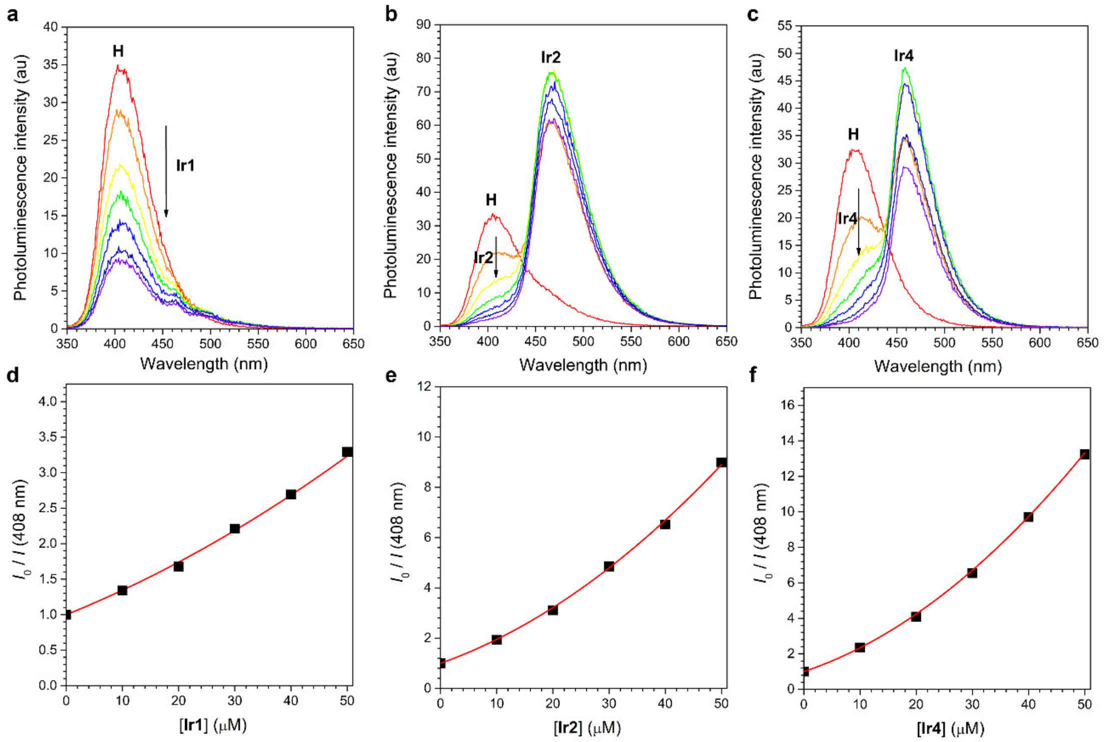

Supplementary Figure 2. Stern–Volmer analyses. a, c, e, Photoluminescence spectra of 100 μM H with added Ir dopant (0–50 μM). Photoexcitation wavelength was 300 nm. **b, d, f,** Stern–Volmer analyses of the fluorescence emission of H (λ_{obs} = 408 nm) in the absence (I_0) and presence (I) of the Ir dopant. The I values were corrected by considering the absorbance of the dopant, following the relationship $I = I_{obs} \times (Abs/Abs_0) \times 1/(1 - 10^{-Abs})$ where I_{obs}, Abs, and Abs_0 are the observed fluorescence intensity, the absorbance at 300 nm in the presence of Ir, and the absorbance at 300 nm in the absence of Ir, respectively. The I_0/I values were fit to the Stern–Volmer equation $I_0/I = (1 + K_a \cdot [\mathrm{Ir}]) \times (1 + k_q \cdot \tau_0 \cdot [\mathrm{Ir}])$. In this equation, K_a, k_q, τ_0, and [Ir] are the association constant, the quenching constant, the fluorescence lifetime of H in the absence of Ir (3.8 ns), and the molar concentration of Ir, respectively. The fit parameters, k_q and K_a, are piled in Supplementary Table 1.

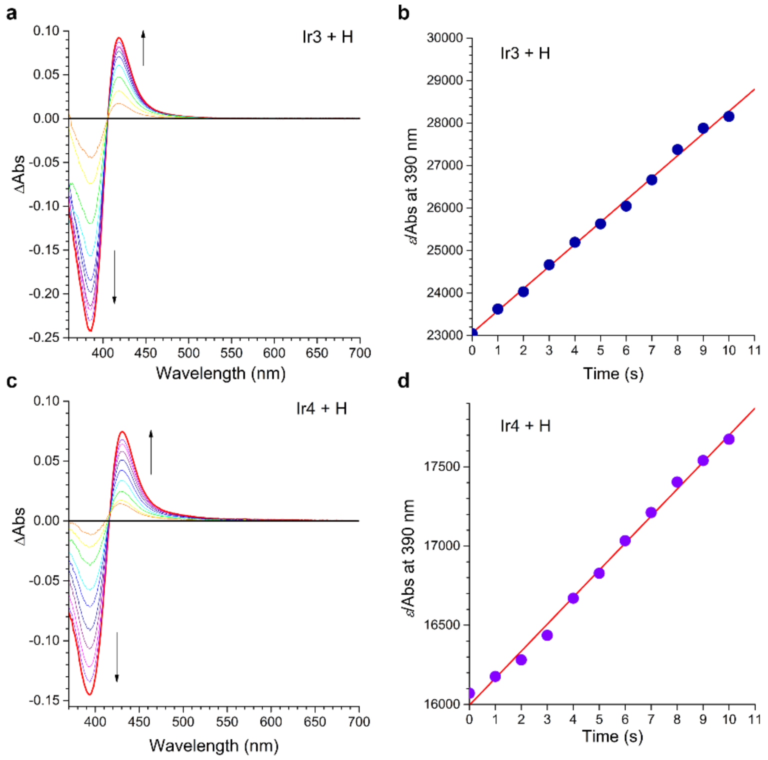

Supplementary Figure 3. Comparison of the rates of photolysis. a, c, UV−vis absorption difference spectra of deaerated THF solutions (3.0 mL) containing 2.5 mM H and 500 μM Ir3 (**a**) or 500 μM Ir4 (**c**) during continuous photoirradiation using a 300 W Xenon lamp for 10 s. **b, d,** Second-order kinetics analyses of the absorption decays at 390 nm (i.e., ε/ΔAbs) of the photolyzed solutions containing 2.5 mM H and 500 μM Ir3 (**b**) or 500 μM Ir4 (**d**). The 390 nm absorption bands correspond to the intraligand charge transfer (ILCT) transition of Ir3 and Ir4. The slopes of the linear fits of the second-order plots correspond to overall rate constants for bimolecular reactions (k_2) between H and Ir. The k_2 values are 522 M^{-1} s^{-1} and 170 M^{-1} s^{-1} for Ir3 and Ir4, respectively. Comparison of the k_2 values demonstrates superior stability of the pair of H and Ir4. The molar absorbance (ε) of Ir3 and Ir4 at 390 nm are 39000 M^{-1} cm^{-1} and 25000 M^{-1} cm^{-1}, respectively. Note that H does not absorb the 390 nm light. Experiments for the pairs of H and Ir1 or Ir2 were unsuccessful due to significant spectral overlaps with the H absorption.

a

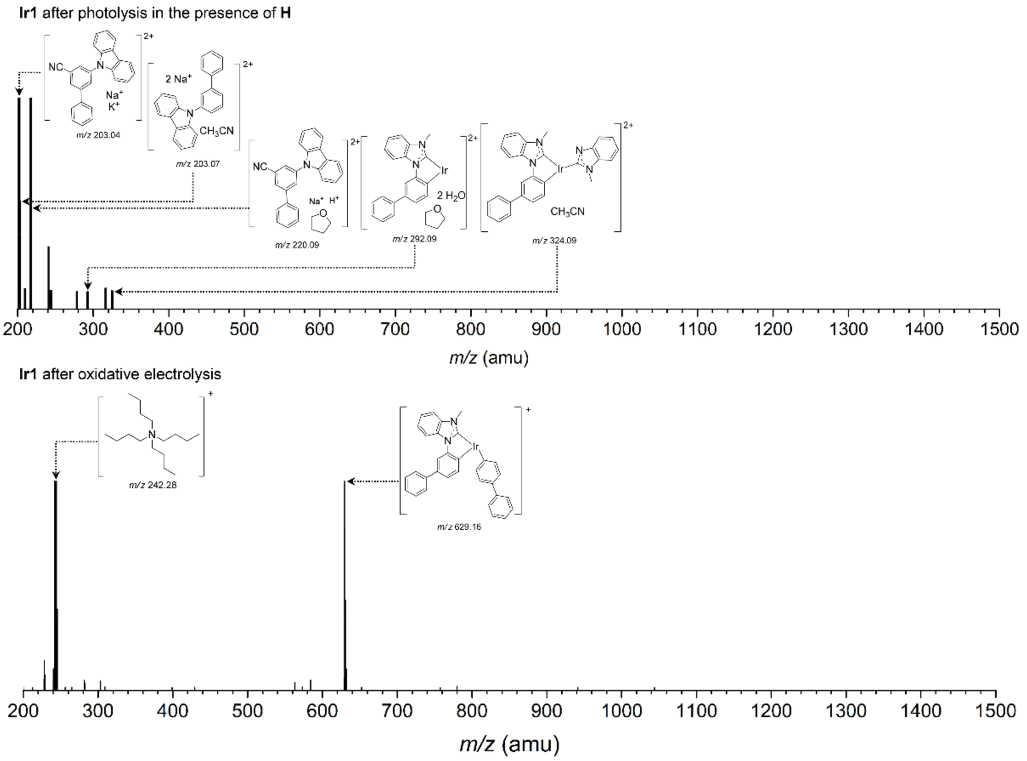
Ir1 after photolysis in the presence of H
m/z 203.04
m/z 203.07
m/z 220.09
m/z 292.09
m/z 324.09
m/z (amu)
Ir1 after oxidative electrolysis
m/z 242.28
m/z 629.16
m/z (amu)

b

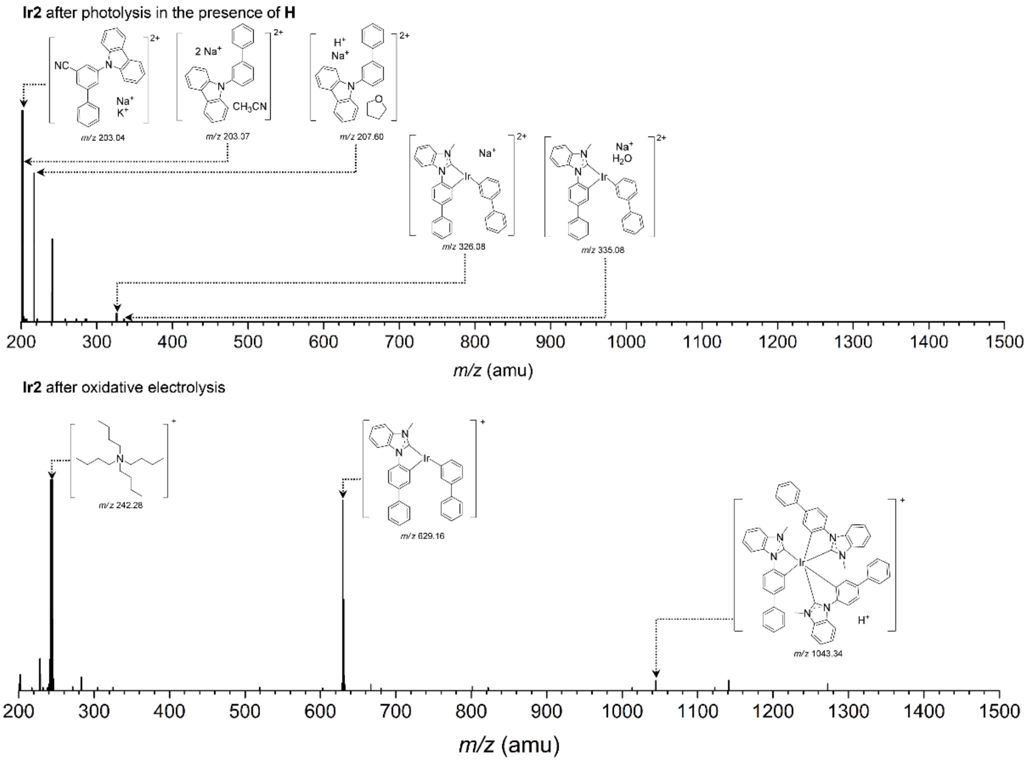
Ir2 after photolysis in the presence of H
m/z 203.04
m/z 203.07
m/z 207.60
m/z 326.08
m/z 335.08
m/z (amu)
Ir2 after oxidative electrolysis
m/z 242.28
m/z 629.16
m/z 1043.34
m/z (amu)

c

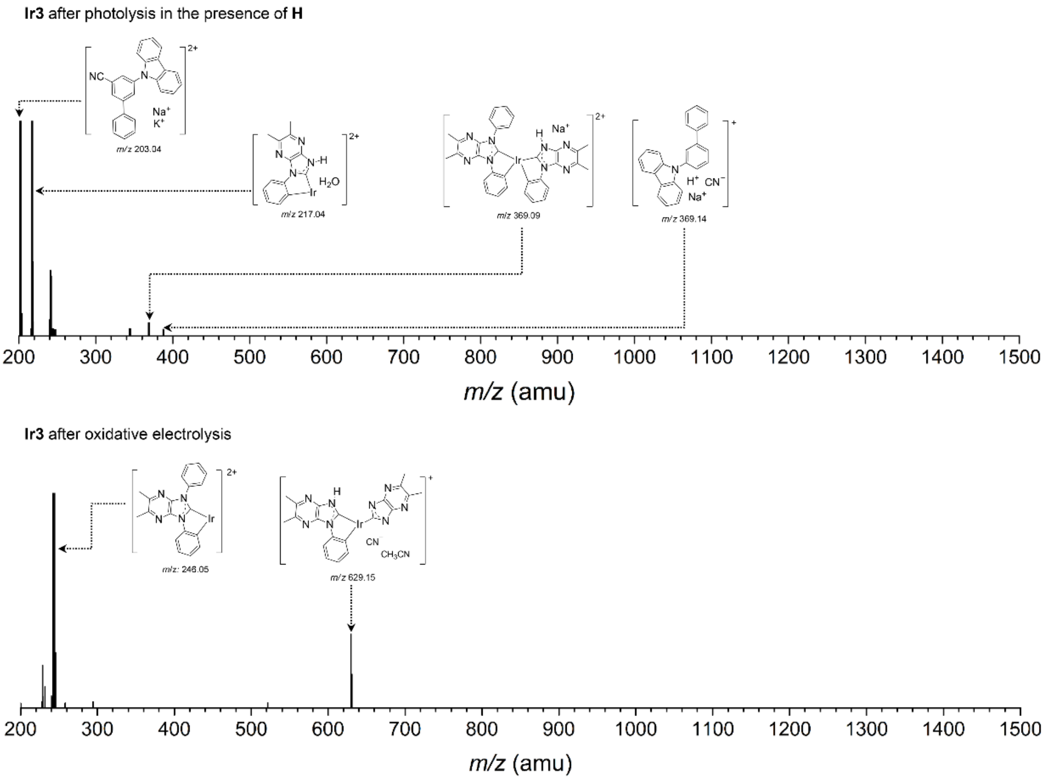

Supplementary Figure 4. Mass analyses of the degradation products in solutions. Electrospray mass spectra (positive mode) taken for THF solutions of Ir1 (**a**), Ir2 (**b**), Ir3 (**c**) after photolysis in the presence of H (300 W Xenon lamp) or oxidative electrolysis (applied potentials: Ir1, 1.0 V vs SCE; Ir2, 1.0 V vs SCE; Ir3, 1.30 V vs SCE). In the case of photolysis, 3.0 mM H was present in each of the solutions. Concentrations of the Ir dopants were 100 μM (photolysis) and 2.0 mM (oxidative electrolysis).

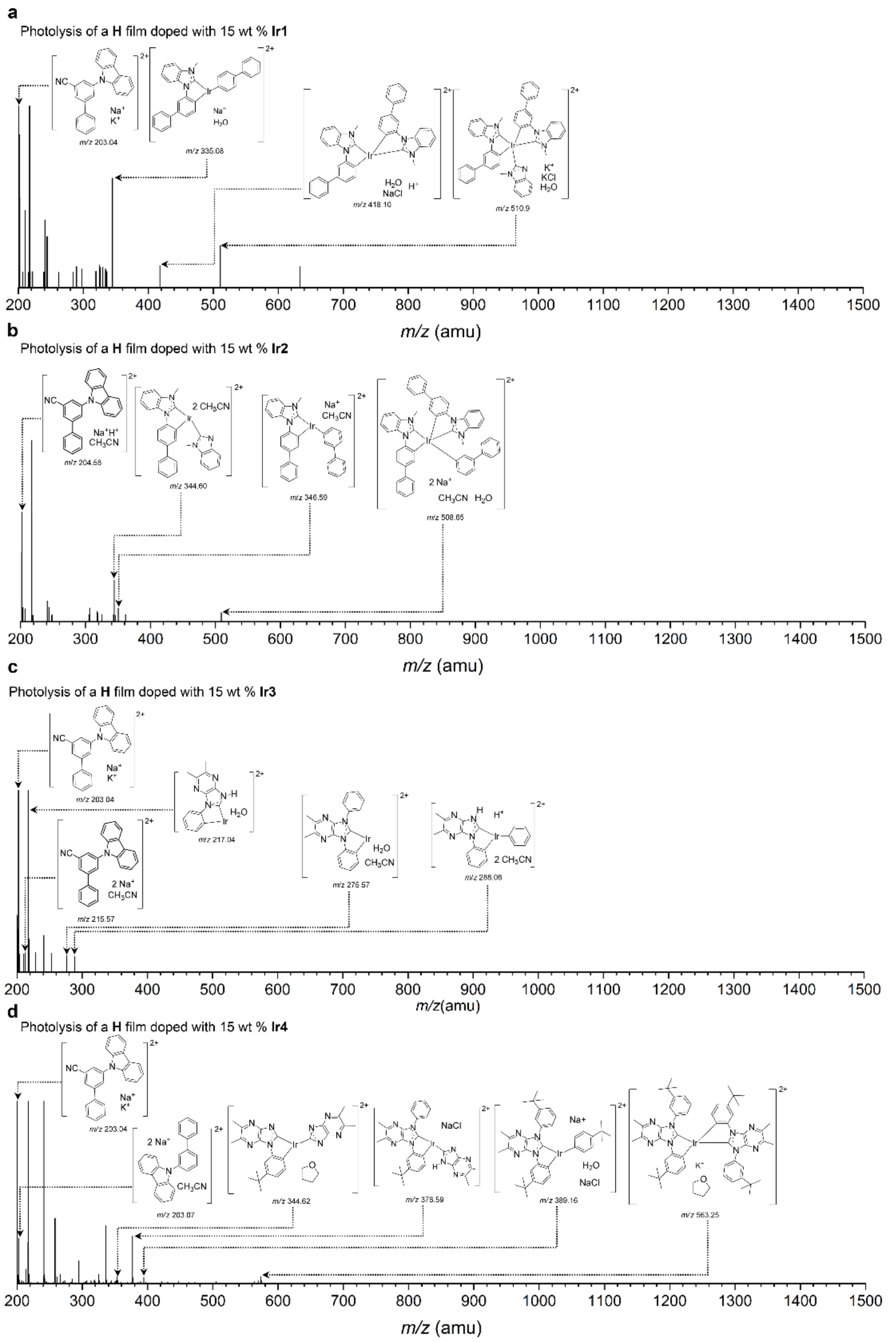

Supplementary Figure 5. Mass analyses of the degradation products in films. Electrospray mass spectra (positive mode) taken for vacuum evaporated films of H doped with 15 wt % Ir1 (**a**), 15 wt % Ir2 (**b**), 15 wt % Ir3 (**c**), 15 wt % Ir4 (**d**) after photolysis (300 W Xenon lamp) for 10 min.

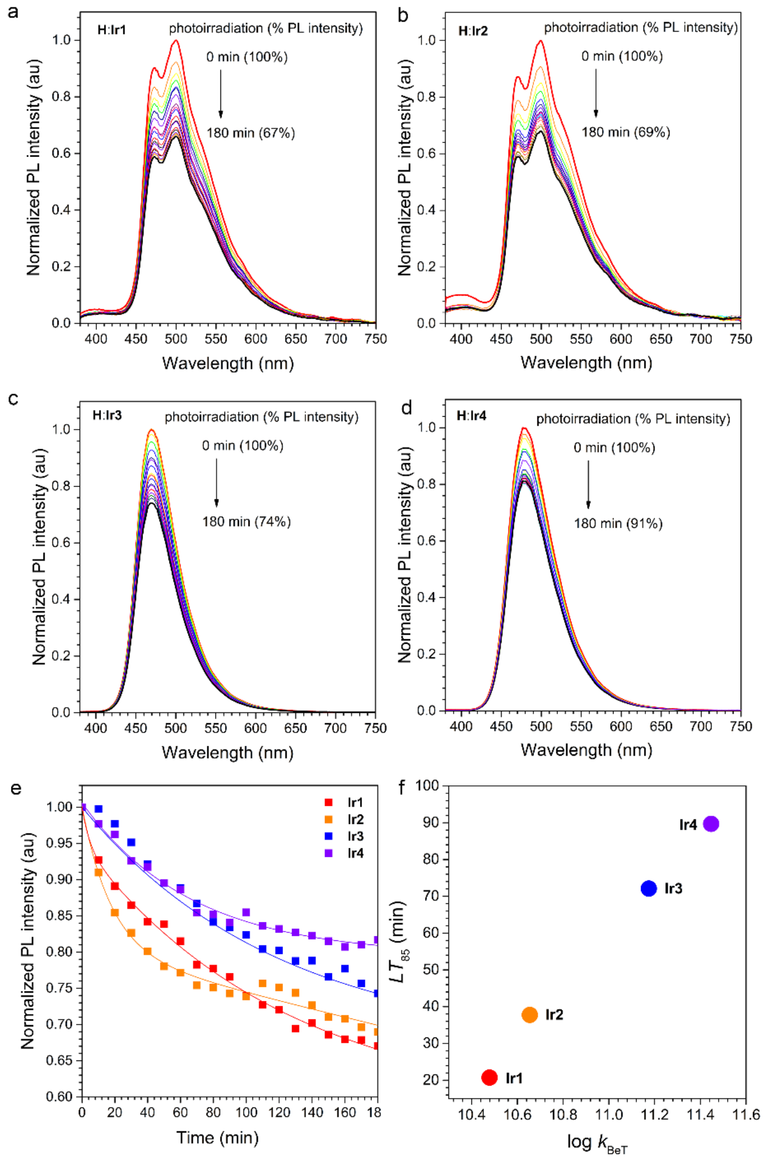

Supplementary Figure 6. Comparison of the photoluminescence decay rates. a–d, Changes in the photoluminescence (PL, excitation wavelength = 325 nm) spectra of the H films doped with 10 wt % Ir1 (**a**), 10 wt % Ir2 (**b**), 10 wt % Ir3 (**c**), or 10 wt % Ir4 (**d**) during continuous photoillumination (325 nm, 3.5 mW, He-Cd laser). **e,** Plots of PL decay traces of the films as a function of photoirradiation times. **f,** A correlation between k_{BeT} and LT_{85}. Here, LT_{85} refers to the time when the normalized PL intensity decreases to 85% of the initial value. The films were encapsulated with glasses under an insert atmosphere to avoid potential degradation by molecular oxygen or moisture.

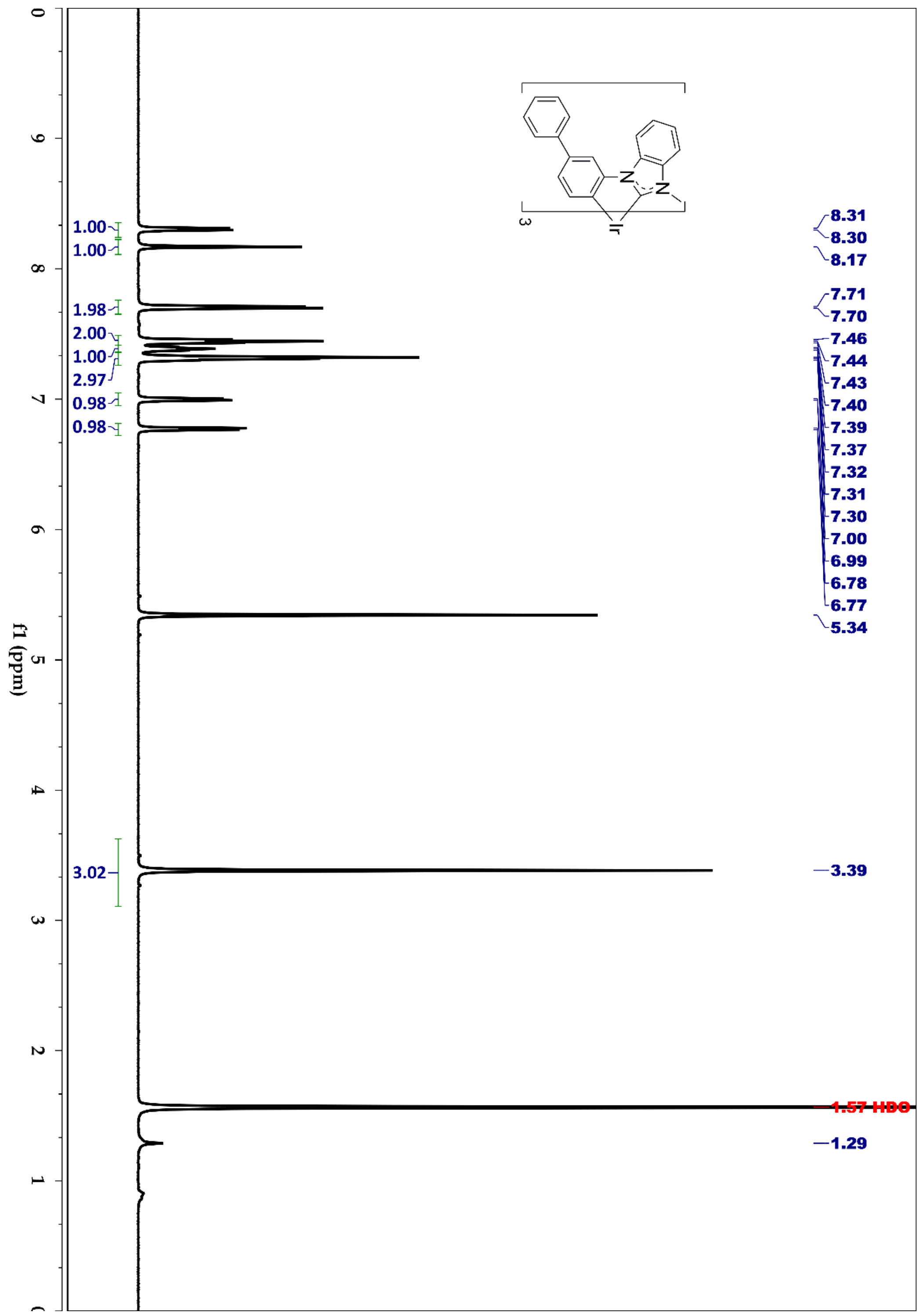

Supplementary Figure 7. ^{1}**H NMR (600 MHz,** CD_2Cl_2**) spectrum of Ir1.**

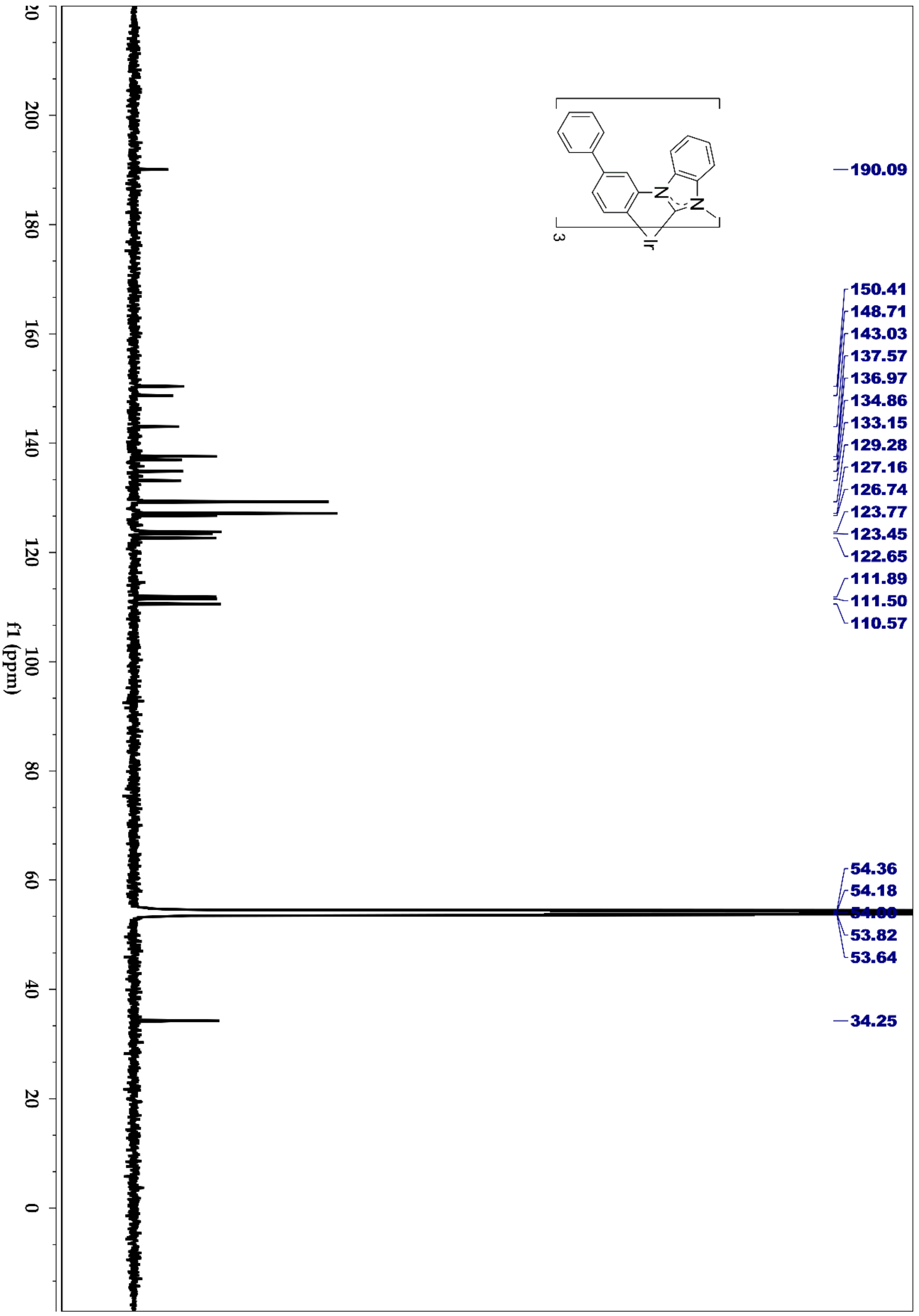

Supplementary Figure 8. **$^{13}C\{^{1}H\}$ NMR (150 MHz, CD_2Cl_2) spectrum of Ir1.**

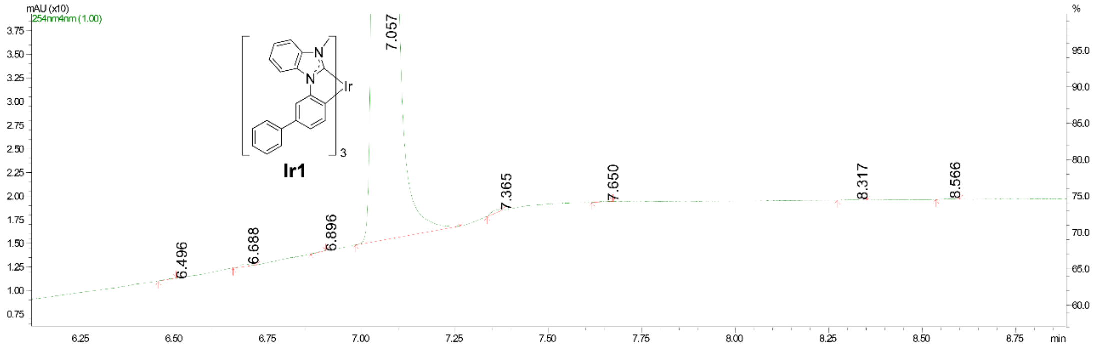

Peak#	Ret. Time	Area	Height	Peak Start	Peak End	Area%
1	6.496	131	-1	6.456	6.504	0.0038
2	6.688	475	251	6.656	6.720	0.0138
3	6.896	131	18	6.864	6.904	0.0038
4	7.057	3434955	1900651	6.984	7.264	99.9540
5	7.365	510	215	7.336	7.376	0.0148
6	7.650	136	64	7.616	7.672	0.0040
7	8.317	95	35	8.272	8.352	0.0028
8	8.566	103	52	8.536	8.600	0.0030

Supplementary Figure 9. High performance liquid chromatogram for Ir1. Purity was determined to be greater than 99.95%.

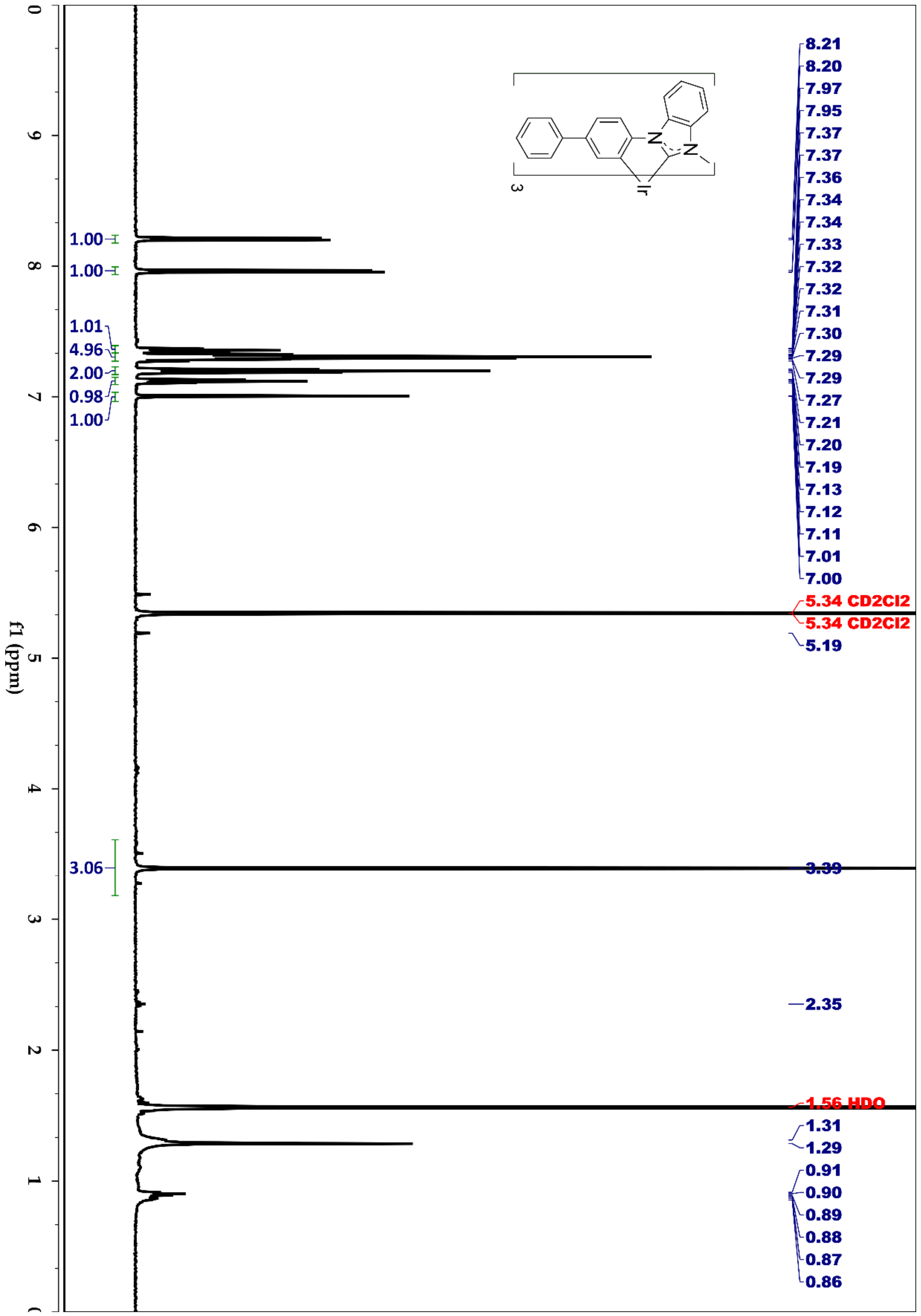

Supplementary Figure 10. ^{1}H NMR (600 MHz, CD_2Cl_2) spectrum of Ir2.

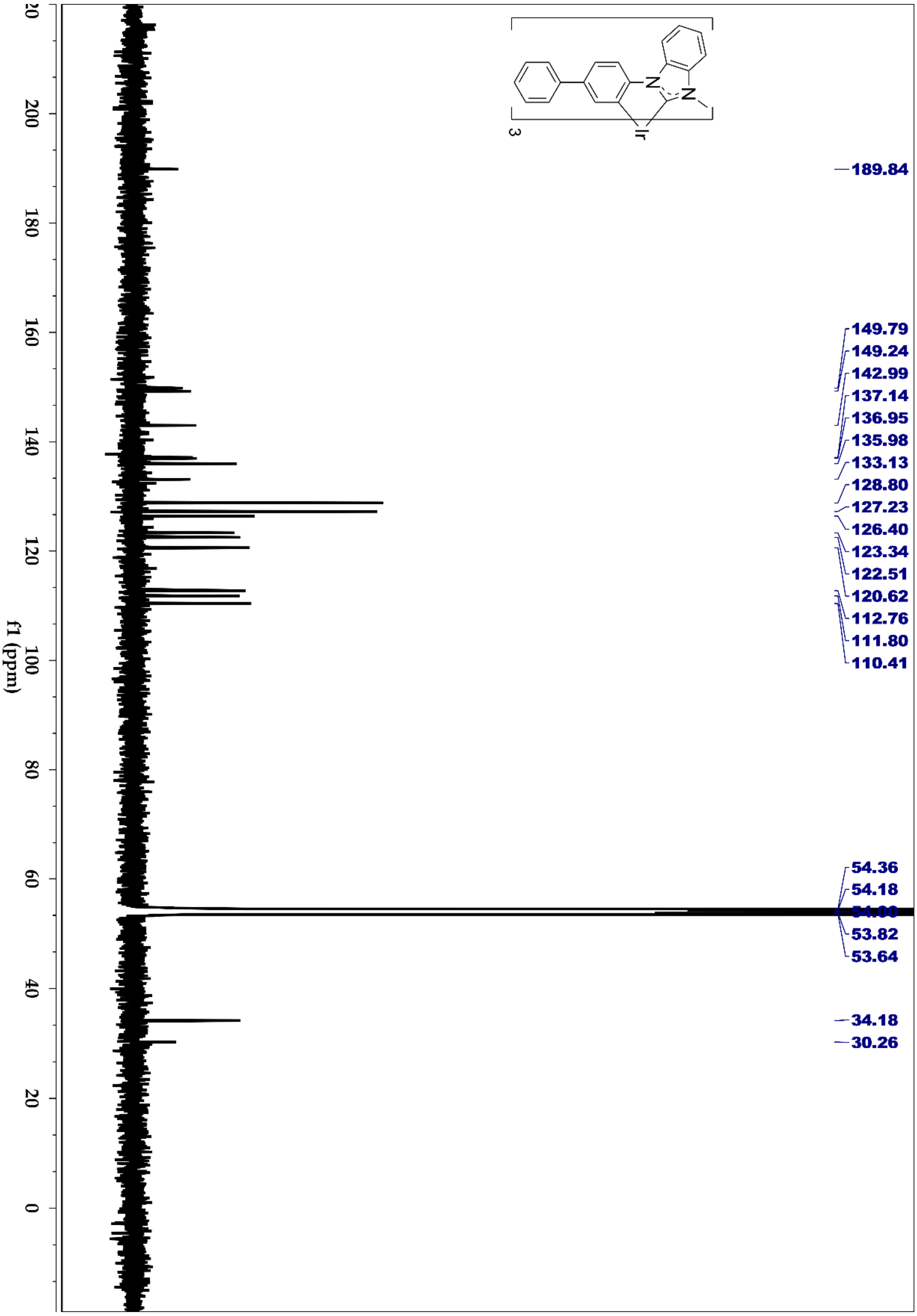

Supplementary Figure 11. $^{13}C\{^{1}H\}$ **NMR (150 MHz,** CD_2Cl_2**) spectrum of Ir2.**

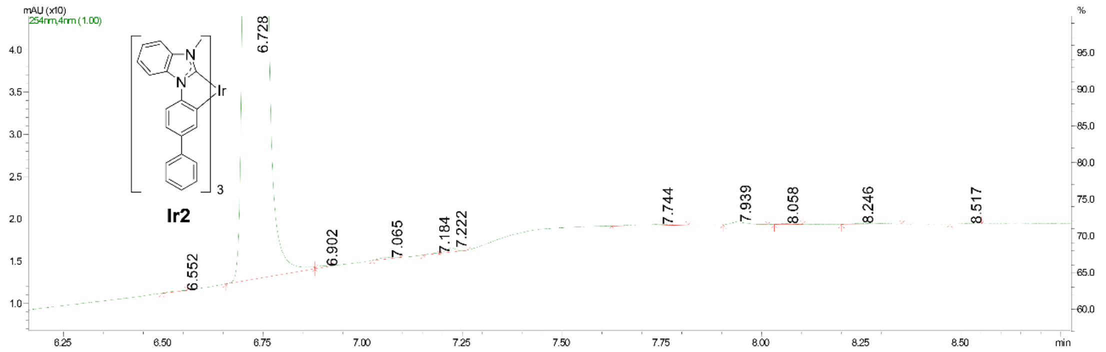

Peak#	Ret. Time	Area	Height	Peak Start	Peak End	Area%
1	6.552	251	26	6.496	6.560	0.0124
2	6.728	2013153	1061400	6.656	6.880	99.7851
3	6.902	371	154	6.880	6.920	0.0184
4	7.065	458	140	7.024	7.096	0.0227
5	7.184	152	41	7.152	7.192	0.0075
6	7.222	851	494	7.192	7.264	0.0422
7	7.744	809	137	7.624	7.816	0.0401
8	7.939	845	311	7.904	8.016	0.0419
9	8.058	193	95	8.032	8.104	0.0096
10	8.246	332	80	8.200	8.352	0.0165
11	8.517	73	33	8.472	8.552	0.0036

Supplementary Figure 12. High performance liquid chromatogram for Ir2. Purity was determined to be greater than 99.79%.

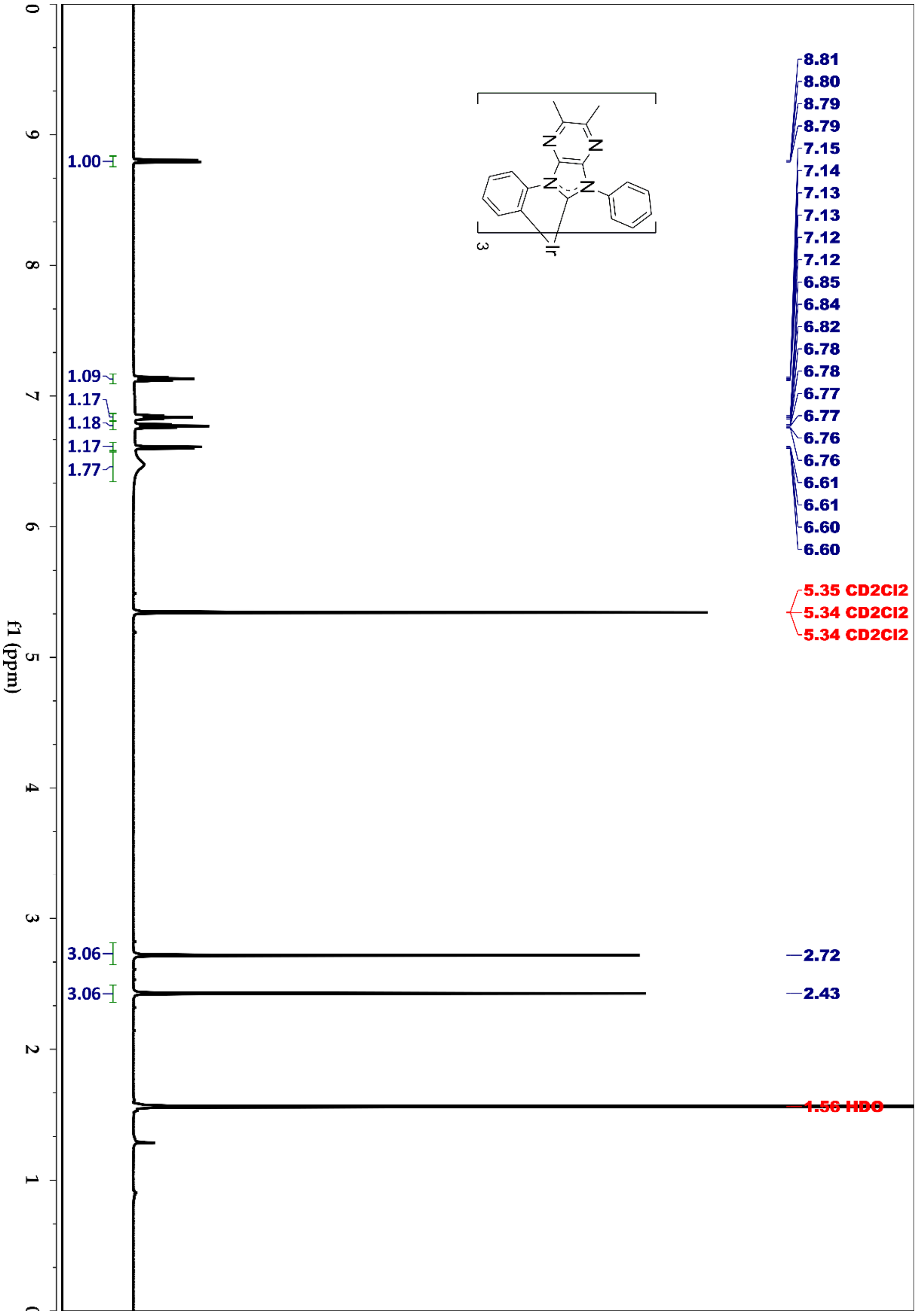

Supplementary Figure 13. **^{1}H NMR (600 MHz, CD_2Cl_2) spectrum of Ir3.**

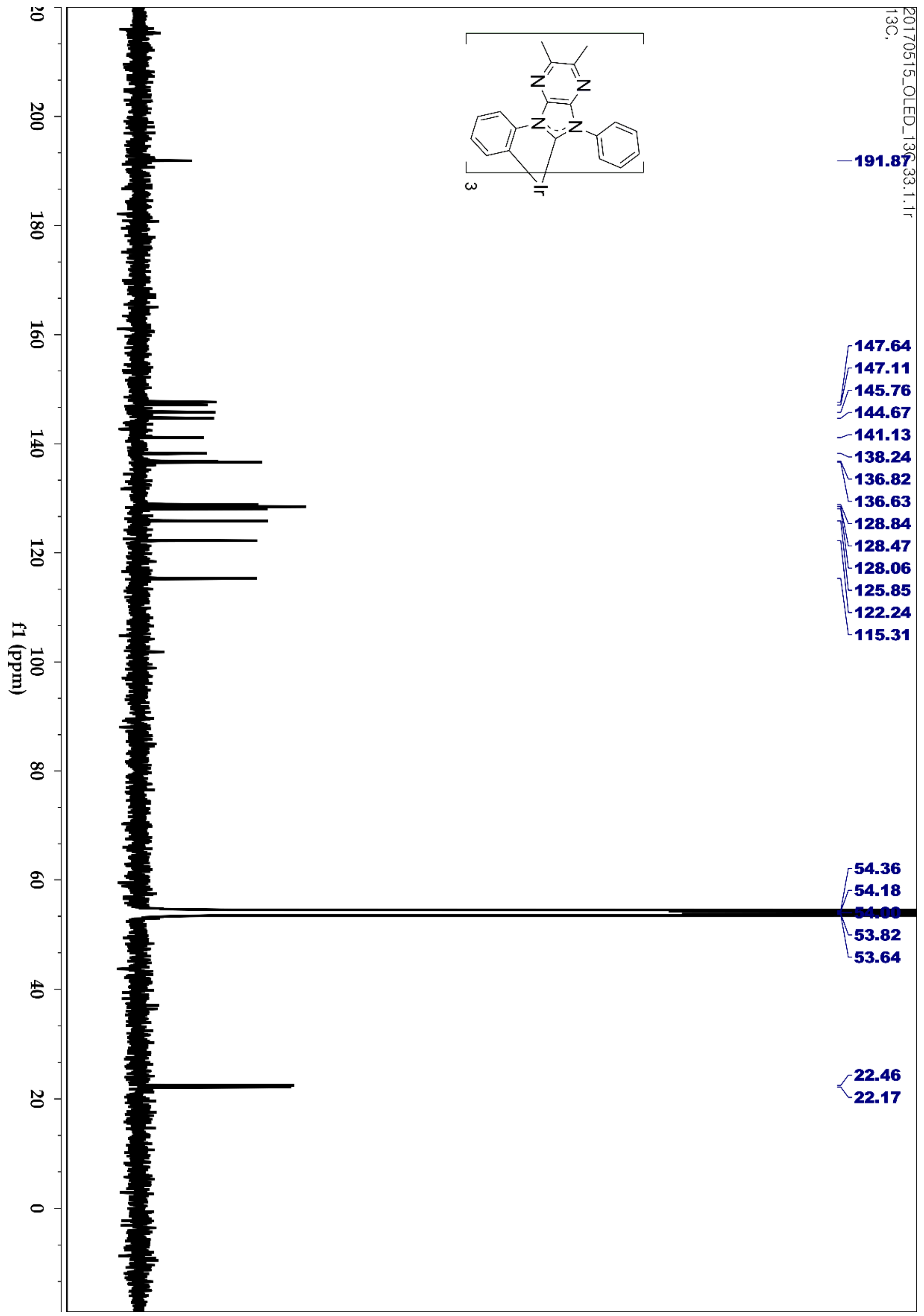

Supplementary Figure 14. $^{13}C\{^1H\}$ NMR (150 MHz, CD_2Cl_2) spectrum of Ir3.

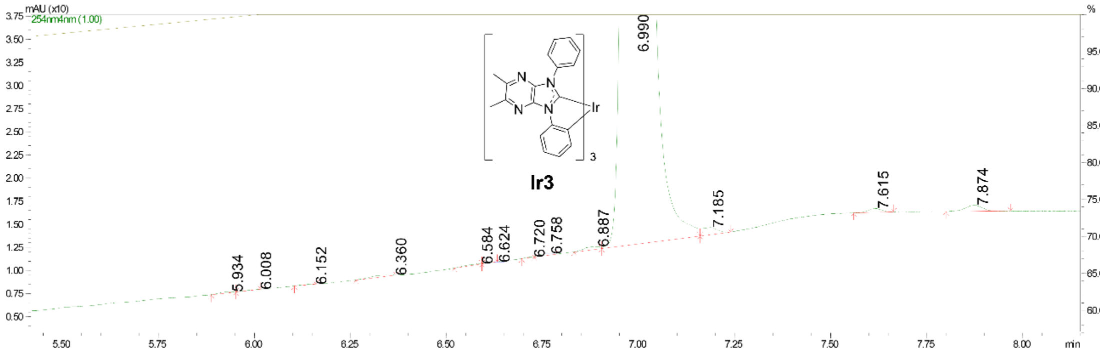

Peak#	Ret. Time	Area	Height	Peak Start	Peak End	Area%
1	5.934	332	106	5.888	5.952	0.0133
2	6.008	137	14	5.952	6.016	0.0055
3	6.152	133	21	6.104	6.160	0.0054
4	6.360	650	31	6.264	6.368	0.0261
5	6.584	354	68	6.520	6.592	0.0142
6	6.624	181	26	6.592	6.632	0.0073
7	6.720	116	49	6.696	6.728	0.0047
8	6.758	480	183	6.728	6.792	0.0193
9	6.887	951	300	6.832	6.904	0.0382
10	6.990	2482201	1038521	6.904	7.160	99.6548
11	7.185	2258	788	7.160	7.240	0.0907
12	7.615	991	401	7.560	7.664	0.0398
13	7.874	2014	662	7.800	7.968	0.0808

Supplementary Figure 15. High performance liquid chromatogram for Ir3. Purity was determined to be greater than 99.65%.

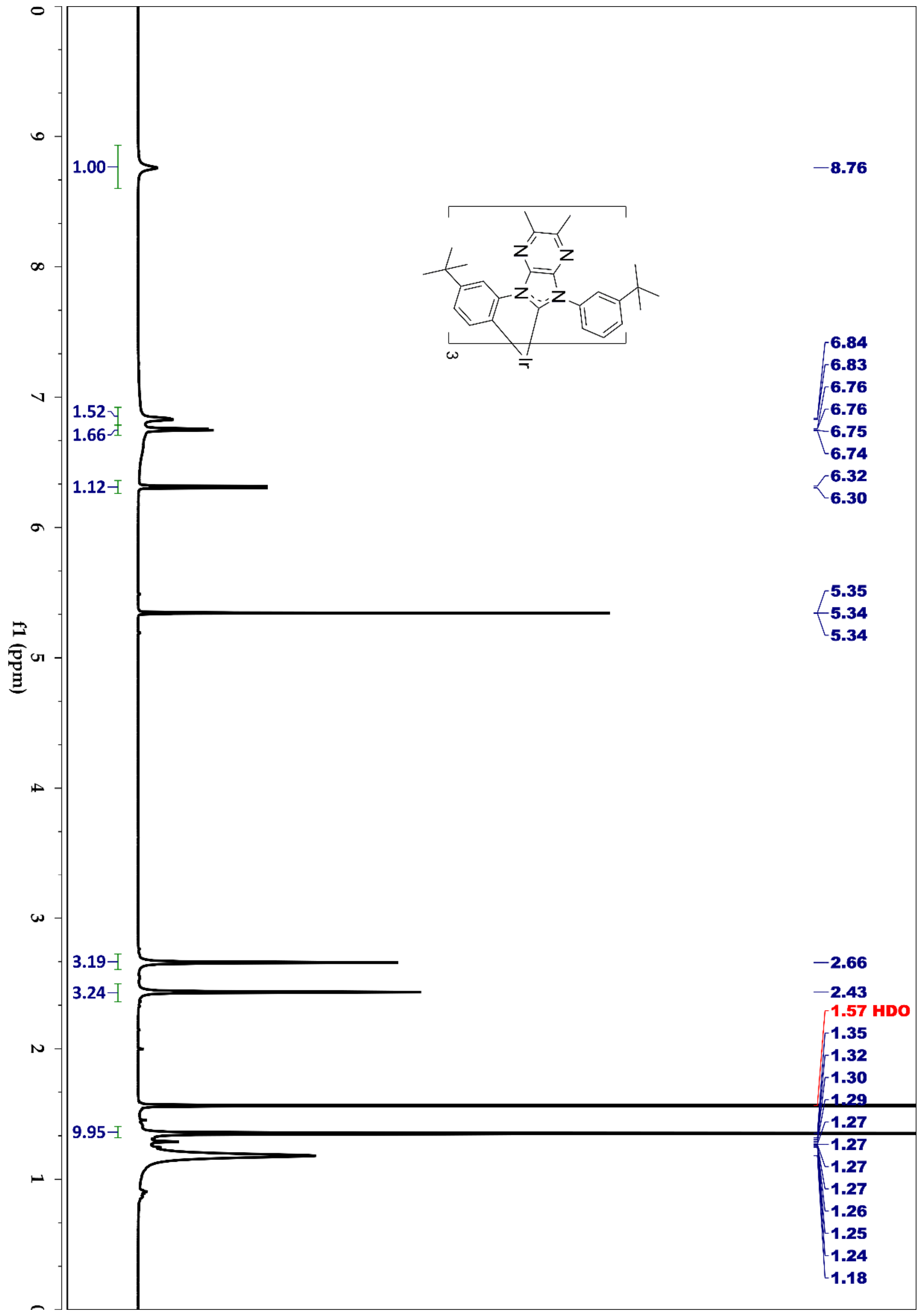

Supplementary Figure 16. ^{1}H NMR (600 MHz, CD_2Cl_2) spectrum of Ir4.

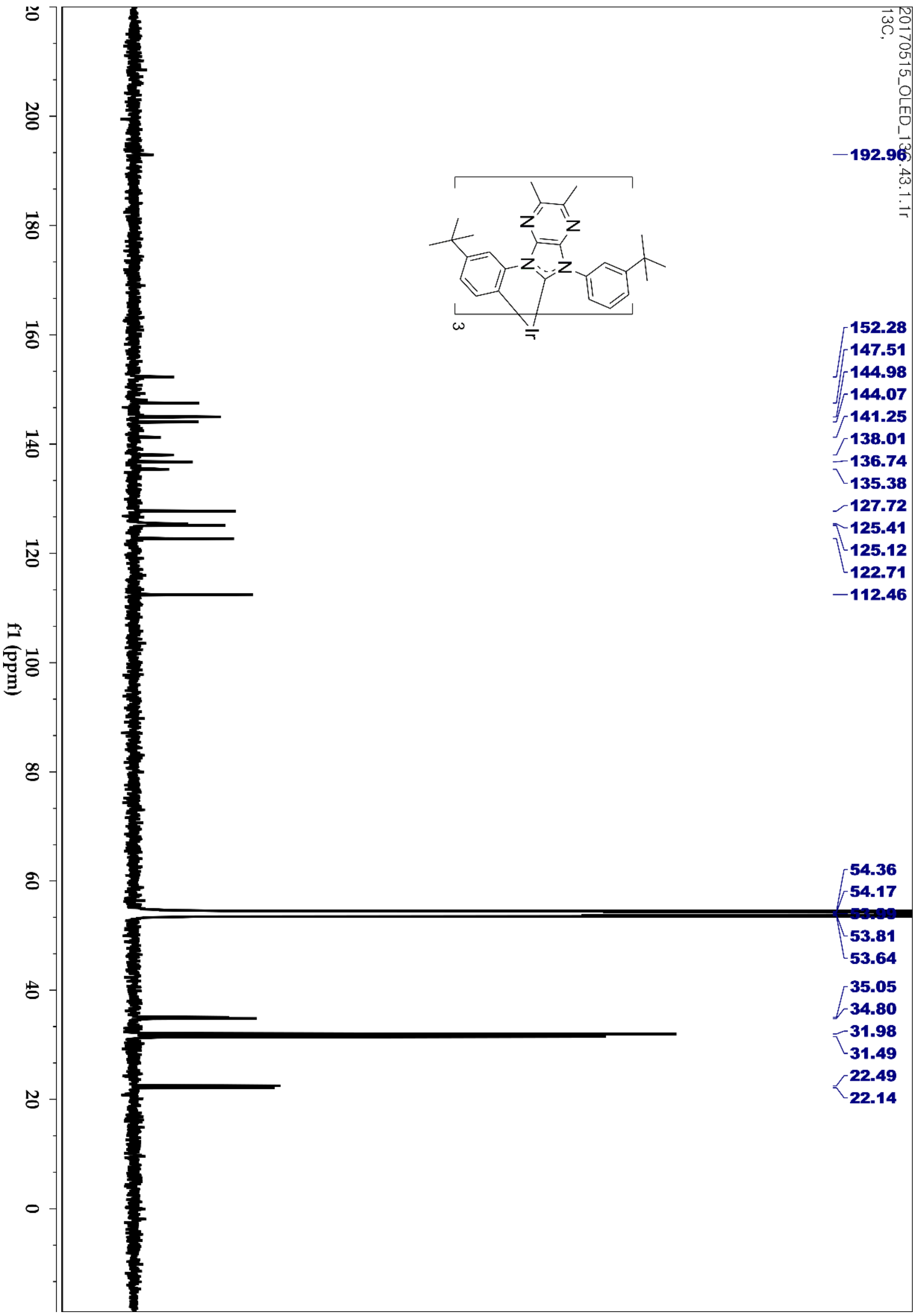

Supplementary Figure 17. $^{13}C\{^{1}H\}$ **NMR (150 MHz, CD_2Cl_2) spectrum of Ir4.**

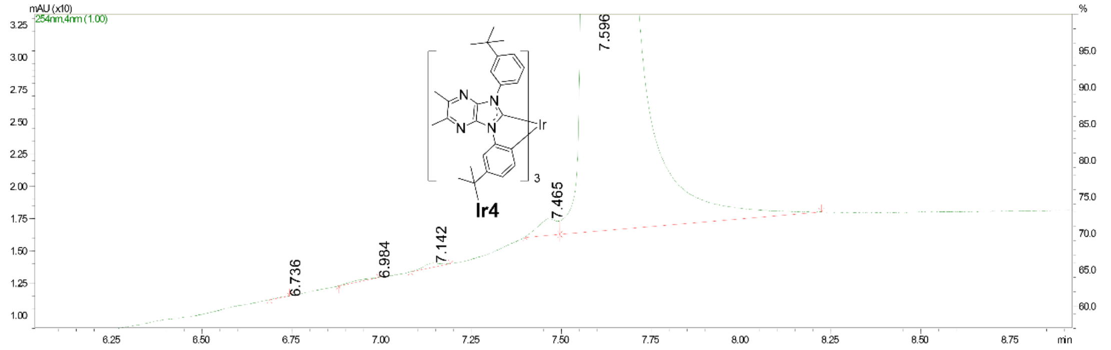

Peak#	Ret. Time	Area	Height	Peak Start	Peak End	Area%
1	6.736	140	8	6.688	6.744	0.0031
2	6.984	586	8	6.880	6.992	0.0128
3	7.142	1101	323	7.080	7.192	0.0241
4	7.465	4914	1270	7.400	7.496	0.1074
5	7.596	4569342	1508878	7.496	8.224	99.8527

Supplementary Figure 18. High performance liquid chromatogram for Ir4. Purity was determined greater than 99.85%.

Supplementary Table 1. The k_q and K_a values obtained from the Stern−Volmer Analyses shown in Supplementary Fig. 2

Dopant	k_q (10^{12} M^{-1} s^{-1})	K_a (10^3 M^{-1})
Ir1	4.2	1.6
Ir2	10	4.0
Ir3	18	6.8
Ir4	14	5.3

Supplementary Table 2. Electroluminescence data for the devices involving emitting layers of H:Ir

Device	Doping concentration (wt %)	V_d [a] (V)	λ_{EL} [b] (nm)	Color coordinates (CIE_x, CIE_y)	EQE_{max} [c] (%)	EQE [d] (%)	LT_{70} [e] (h)
H:Ir1	10	7.31	470	(0.186, 0.338)	2.0	0.5	0.92
	20	6.55	470	(0.187, 0.349)	2.8	0.7	1.88
H:Ir2	10	6.28	467	(0.196, 0.350)	4.1	1.3	1.89
	20	5.59	468	(0.200, 0.366)	6.9	2.6	4.11
H:Ir3	10	4.11	466	(0.139, 0.165)	14.3	11.6	12.27
	20	3.91	466	(0.140, 0.176)	16.0	13.9	19.70
H:Ir4	10	3.94	473	(0.146, 0.246)	17.9	17.1	60.19
	20	3.86	475	(0.149, 0.264)	18.2	17.8	93.05

[a]Driving voltage at 500 cd m^{-2}. [b]Peak wavelength. [c]Maximum EQE. [d]EQE determined at 500 cd m^{-2}. [e]Operation time when the luminance decreases to 70% of its initial value. Constant current mode.

Supplementary Table 3. Electroluminescence data for the devices involving emitting layers of mCBP:Ir

Device	Doping concentration (wt %)	V_d [a] (V)	λ_{EL} [b] (nm)	Color coordinates (CIE_x, CIE_y)	EQE_{max} [c] (%)	EQE [d] (%)	LT_{70} [e] (h)
mCBP:Ir1	10	7.24	467	(0.167, 0.216)	6.0	1.6	0.68
	20	6.65	468	(0.170, 0.259)	8.5	1.6	0.33
mCBP:Ir2	10	7.19	466	(0.177, 0.256)	8.0	3.1	1.04
	20	7.21	467	(0.179, 0.273)	9.5	3.0	0.43
mCBP:Ir3	10	4.98	463	(0.138, 0.141)	20.0	18.2	6.88
	20	4.37	463	(0.139, 0.152)	17.4	16.9	11.34
mCBP:Ir4	10	5.53	471	(0.142, 0.217)	15.9	18.0	17.50
	20	4.77	473	(0.145, 0.238)	16.9	16.9	44.66

[a]Driving voltage at 500 cd m^{-2}. [b]Peak wavelength. [c]Maximum EQE. [d]EQE determined at 500 cd m^{-2}. [e]Operation time when the luminance decreases to 70% of its initial value. Constant current mode.

Supplementary Table 4. Electrochemical potentials of H and mCBP

Host	λ_{em} (nm)	E_{ox} (V vs SCE)	E_{red} (V vs SCE)	E^*_{red} (V vs SCE)
H	411	1.50	−1.82	1.28
mCBP	346	1.48	−1.79	1.88

Supplementary Reference

(1) Ihn, S.-G., Lee, N., Jeon, S. O., Sim, M., Kang, H., Jung, Y., Huh, D. H., Son, Y. M., Lee, S. Y., Numata, M., Miyazaki, H., Gómez-Bombarelli, R., Aguilera-Iparraguirre, J., Hirzel, T., Aspuru-Guzik, A., Kim, S. & Lee, S. An alternative host material for long-lifespan blue organic light-emitting diodes using thermally activated delayed fluorescence. *Adv. Sci.* 1600502 (2017).
